# Research progress on black phosphorus hybrids hydrogel platforms for biomedical applications

**DOI:** 10.1186/s13036-023-00328-w

**Published:** 2023-01-30

**Authors:** Hao-xuan Li, Kun-chi Zhao, Jia-jia Jiang, Qing-san Zhu

**Affiliations:** Department of Spine Surgery, China-Japan Union Hospital of Jilin University, N.126 Xiantai Street, Changchun, 130033 Jilin People’s Republic of China

**Keywords:** Black phosphorus (BP), Hybrid hydrogels, Nanomaterials, Biomedical applications, Tissue engineering

## Abstract

Hydrogels, also known as three-dimensional, flexible, and polymer networks, are composed of natural and/or synthetic polymers with exceptional properties such as hydrophilicity, biocompatibility, biofunctionality, and elasticity. Researchers in biomedicine, biosensing, pharmaceuticals, energy and environment, agriculture, and cosmetics are interested in hydrogels. Hydrogels have limited adaptability for complicated biological information transfer in biomedical applications due to their lack of electrical conductivity and low mechanical strength, despite significant advances in the development and use of hydrogels. The nano-filler-hydrogel hybrid system based on supramolecular interaction between host and guest has emerged as one of the potential solutions to the aforementioned issues. Black phosphorus, as one of the representatives of novel two-dimensional materials, has gained a great deal of interest in recent years owing to its exceptional physical and chemical properties, among other nanoscale fillers. However, a few numbers of publications have elaborated on the scientific development of black phosphorus hybrid hydrogels extensively. In this review, this review thus summarized the benefits of black phosphorus hybrid hydrogels and highlighted the most recent biological uses of black phosphorus hybrid hydrogels. Finally, the difficulties and future possibilities of the development of black phosphorus hybrid hydrogels are reviewed in an effort to serve as a guide for the application and manufacture of black phosphorus -based hydrogels.

**Graphical Abstract:**

Recent applications of black phosphorus hybrid hydrogels in biomedicine.
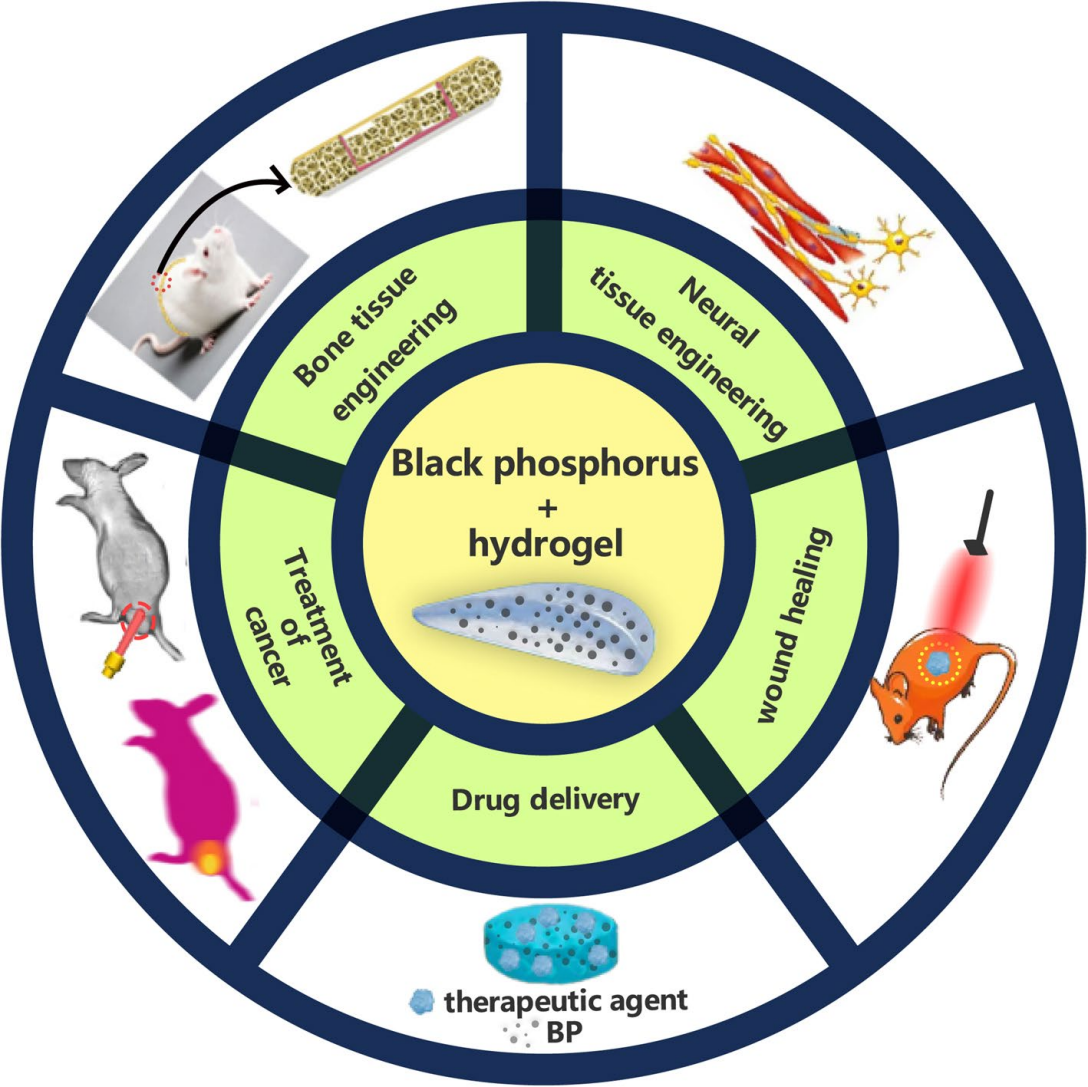

## Introduction

Hydrogels resembling the tissue structure of natural extracellular matrix are among the most well-established materials for biomedical applications. Hydrogels are defined as three-dimensional (3D) networks with hydrophilic groups cross-linked by natural and/or synthetic polymers such that they absorb and retain a significant volume of liquid components while keeping their shape and 3D structure after swelling [1, 2]. The highly hydrous 3D porous network of hydrogels can imitate the microenvironment of natural tissues. Hydrogels are often engineered to retain, release, or trap materials, which have been applied to many biomedical fields including tissue engineering, wound healing, medication administration, bio-imaging, and diagnostics [3, 4]. For the preparation of hydrogels, it is necessary to choose materials based on a range of essential properties, including swelling, mechanical properties, diffusion rate, and chemical functionality. These properties are dependent on the cross-link density, the distance between cross-links, the gel’s macromolecular structure, and the residual chemicals (such as monomers, initiators, and others substances) [5]. Traditional hydrogels have mediocre mechanical characteristics and unspectacular characteristic functions, limiting their potential applications. The fact that nanocomposite hydrogels combine the properties of polymers and nanoparticles to provide excellent functionalities draws greater attention to them. Among the nano-fillers of many hydrogels, two-dimensional (2D) nanomaterials play an increasingly important role [6]. Black phosphorus is an outstanding example due to its biodegradability, biocompatibility, photothermal performance, electrical conductivity, and high surface area mechanical strength [7, 8]. As with other nanocomposite hydrogels, the preparation of black phosphorus-hybrid hydrogels includes blending method, grafting method, in-situ precipitation, and freeze-thaw method [9, 10]. The use of physical crosslinking, chemical crosslinking, and the principle of electrostatic interactions in aforementioned methods will be discussed further in the following section, along with their biomedical applications.

## Synthesis and properties of black phosphorus

In the last decade, novel 2D nanomaterials with diverse characteristics have garnered exploding interest in the domains of biomedicine and other disciplines, where they play an increasingly crucial role [11–13]. These single or multilayer nano-crystalline materials with planar shape exhibit greater interatomic interactions than stacking interactions. More than a thousand 2D material structures have been found and categorized into a variety of monolayer or multilayer structures, each of which exhibits unique physicochemical properties. They cover the complete periodic table, from transition metals through boron −/ carbon −/ nitrogen −/ sulfur groups, as well as graphene and its derivatives (e.g., graphene oxide or reduced graphene oxide, carbon nano tube), single element materials (e.g., Xene materials including phosphorene, silicene, germanene), metal carbides and nitrides (e.g., MXenemarterials including Ta_4_C_3_ Tx), transition metal disulphide (TMDCs materials including MoS_2_, WS_2_, WSe_2_), transition metal oxides (TMOs including TiO_2_, MnO_2_), layered double hydroxide (LDH), hexagonal boron nitride (hBN), graphite carbon nitride (gC_3_N_4_) or silicate clay (nanoclay), and other materials [7, 14, 15]. Due to their distinctive shape, 2D nanomaterials possess a huge surface area and anisotropic physical/chemical properties in addition to distinct mechanical/chemical/optical properties, biocompatibility, and degradability. However, several of these materials have intrinsic limitations, such as graphene’s lack of band gap and MoS_2_’s very poor carrier mobility [14–17]. An ideal 2D material should have a high charge mobility and a sufficient and tunable bandgap, and the emergence of black phosphorus satisfies these scientific conditions. This band structure provides a much-needed gap for field-effect transistor (FET) applications in two-dimensional materials such as graphene, while the thickness-dependent direct band gap, which permits wide-band absorption from the visible to mid-infrared range, could lead to applications in optoelectronics, particularly in the infrared field. In addition, black phosphorene can offer the optimal trade-off between mobility and switching current ratio, which is highly desirable for the development of high-speed, flexible electronic systems that can function in the tens of megahertz frequency range [16].

**Fig. 1:**
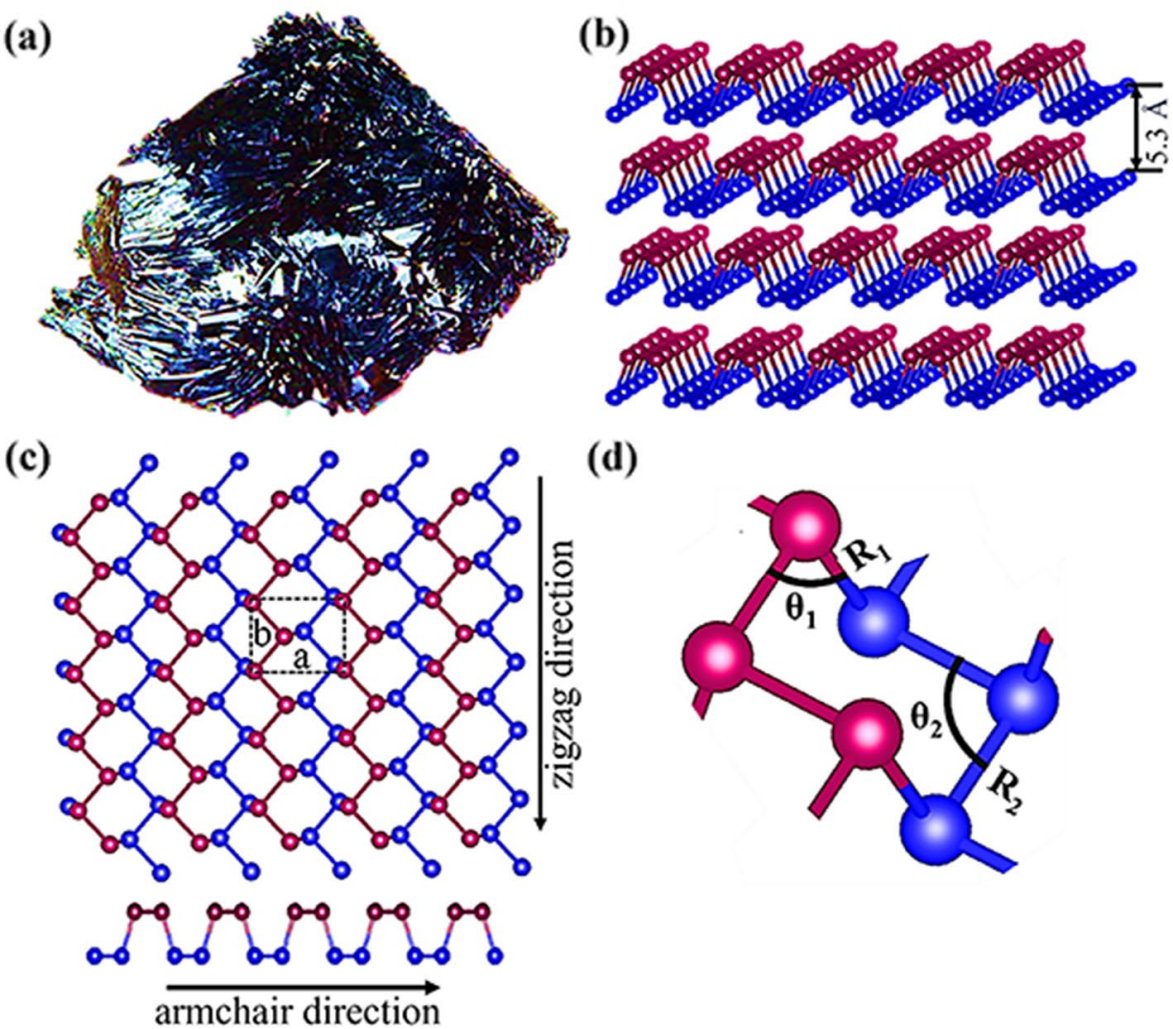
(**a**): image of bulk BP crystal; (**b**) Perspective side view of multi-layer BP; (**c**) Top and side views of single-layer phosphorene where ‘a’ and ‘b’ represent lattice parameters; (**c**) A representation of the arrangement of atomic structures, illustrating dihedral/hinge angles and bond lengths, where different colored P atoms in each image represent P atoms in different planes. Reproduced with permission from Ref [16]. 2019IOP Publishing Ltd. All rights reserved.

Phosphorene, sometimes referred to as black phosphorus (BP) when layered, is a 2D substance. Since Bridgman successfully obtained the first massive BP crystals in 1914, there has been little interest in BP research for over a century. Since two independent teams successfully stripped BP of its monolayer in 2014 [17, 18], however, researchers have been uncovering its unique combination of properties, such as high surface activity, adjustable band gap, favorable on/off current ratio, excellent carrier mobility, near-infrared photo responsiveness (NIR), biocompatibility, and non-toxic biodegradation products [19–21]. These properties cannot be separated from those of its fundamental structure (see Fig. 1). Phosphorus is one of the most plentiful elements in the earth’s crust, making up around 0.1% of the total volume [22]. Phosphorus occurs primarily as four allotropes: white phosphorus (WP), red phosphorus (RP), violet phosphorus (VP), and black phosphorus (BP). Under ambient circumstances, WP is the most reactive and unstable allotrope, whereas BP is the most stable and least reactive [23, 24]. BP is a kind of highly purified black sheet basic crystal. Chemical bonds bind the phosphorus atoms in BP to three phosphorus atoms in the vicinity. The several BP layers are joined by van der Waals’ force. BP has a layer-dependent band gap that is adjustable from 0.3 eV (bulk) to 2.0 eV (monolayer) and has stronger absorption in the UV and NIR areas than the 2D materials discussed before. Also shown modest carrier mobility is BP. BP has been developed as saturable absorbers for solid-state lasers to create short pulses because to its high potential for pulse generation in the 3 μm spectral region of the mid-infrared [25]. The excellent biocompatibility and biodegradability of BP in vivo imply that it is more likely than other materials to be acceptable for interdisciplinary biological applications [8, 26, 27]. Due to edge states and quantum confinement phenomena, BP nanoribbons and zero-dimensional quantum dots with unique properties can be produced by morphological engineering. BP quantum dots were produced by ultrasonic liquid phase stripping, solvent heat treatment, and electrochemical stripping on 2D BP nanosheets. BPQD features an extremely tiny dimension, a broad band gap that is tunable, good edge states, and a greater surface area to volume ratio [28].

The combination of these prosperities makes BP and its derivatives attractive and promising solutions for biological applications when used as a nano-filler of hydrogels to form a system.

**Fig. 2:**
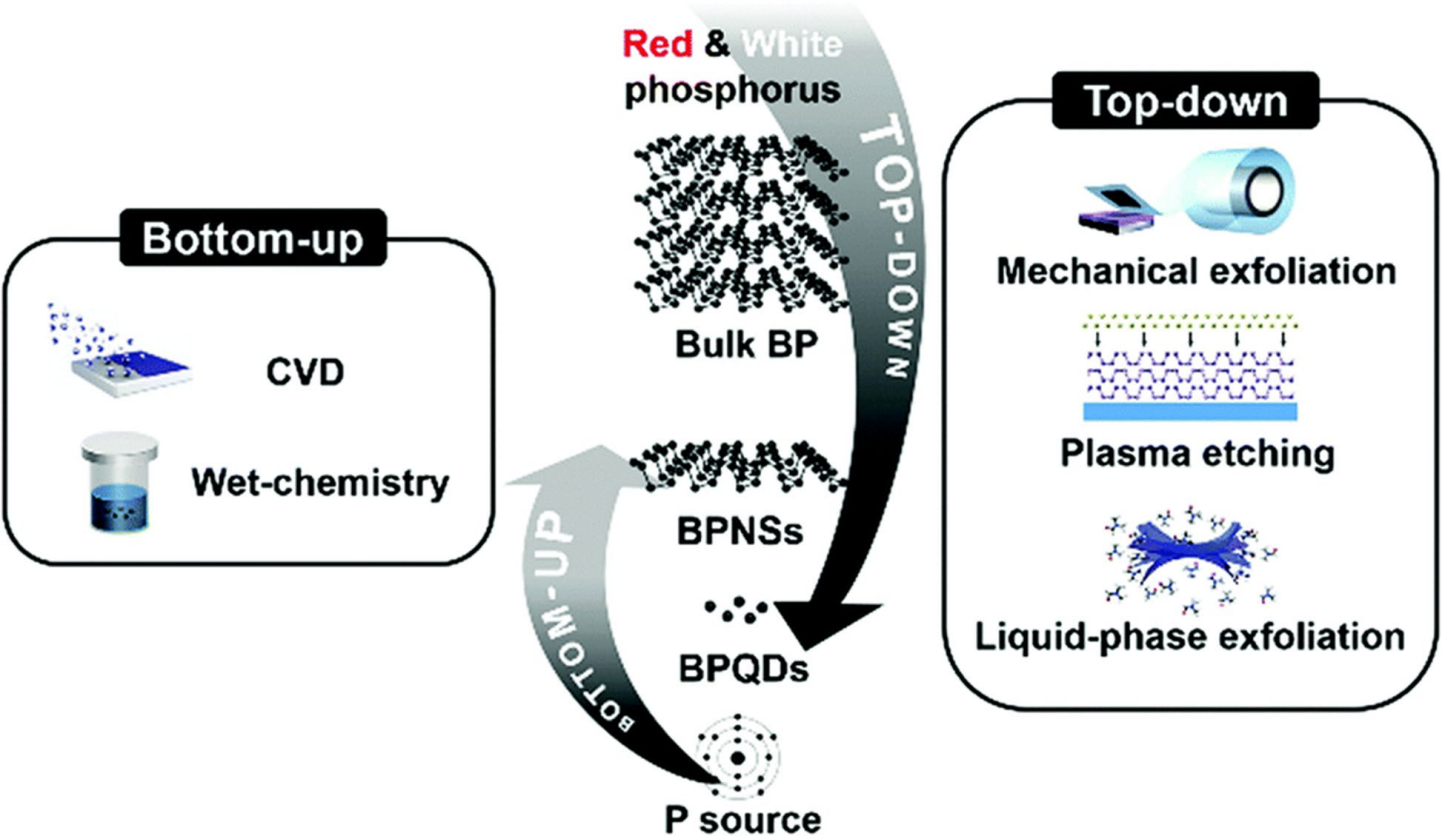
Scheme of the synthetic method for nano-BP .Reproduced with permission from Ref [26]. Copyright 2019Wiley Ltd. All rights reserved

Due to the recent invention of technologies to peel bulk BP powders or crystals into nanostructures, its promise for biological applications has only been examined in the last decade. The fabrication of ultra-thin 2D nanomaterials is often separated into two fundamental methods, the “top-down approach” and the “bottom-up approach,” and the preparation of BP is no exception. Typically, top-down approach concentrates on the exfoliation of bulk materials with the aid of a driving force (e.g., mechanical exfoliation, chemical exfoliation, and liquid exfoliation), resulting in the disintegration of the material into nanoparticles. The approach comprises chemical synthesis and extraction from certain starting materials. Top-down approach typically consist of mechanical exfoliation, liquid exfoliation, plasma-assisted techniques such as plasma etching, ultrasound-assisted exfoliation, and electrochemical techniques [29, 30] (see Fig. 2). It is highly recommended to read the following reviews on the synthesis of black phosphorus [26, 31–35]. The hunt for ever-simpler and economic mass synthetic production technologies will usher in an expansion of applications.

## Biomedical application of black phosphorus hybrids hydrogel

Despite the fact that research on BP started in 2014, the research results of this novel 2D material has achieved a resounding success. This section illustrates the significance of BP in biological applications such as bone tissue engineering (see Table 1), nerve tissue engineering, immunological and cancer treatment, skin wound healing, and drug delivery.

**Table 1:** Application of black phosphorus composite hydrogel in bone tissue engineering

Hydrogel matrix	Black phosphorus form	Scaffold performance	Animal model	References
GelMA and U-Arg-PEA	BPNs	Strong mechanical properties, capturing calcium ions, accelerating biomineralization, and enhancing osteogenic differentiation of HDPSCS	Rabbit skull defect model	[36]
GEL and DFO	BPNs	Excellent swelling, degradation and release rate, satisfactory biocompatibility, capable of promoting the proliferation and osteogenesis of hBMSC in vitro, improving bone regeneration and neovascularization in vivo	Rat model of acute ischemic tibial defect	[37]
GEL	BPNs	Excellent NIR photothermal antibacterial, eliminating cancer cell properties, and enhancing bone regeneration	Rat model of skull defect	[38]
Agarose	BPNs	Providing phosphorus source and nucleation site, accelerating PO _4_^3−^ and Ca ^2+^ reactions to promote biomineralization; The mechanical properties and bio-mineralization capabilities are customized by adjusting the timing and location of NIR light	–	[39]
Chitosan/ collagen	MSC membrane coated BPNs	Activating the heat shock response of osteoblasts, stimulating downstream responses, enhancing osteoblast migration/differentiation, and stimulating biomineralization processes to promote bone healing upon remote NIR activation	Rat model of skull defect	[40]
PLGA	BPNs	Strategies for heat-stimulated bone regeneration, ranging from inefficient external hyperthermia to more effective self-hyperthermia with “smart” bone implants under remote control	Rat model of tibial defect	[41]
CNTpega -OPF	BPNs	Injectable, good conductivity combined with electrical stimulation, improving the adhesion, proliferation, filament and focal adhesion development, and osteogenic differentiation of pre-osteoblast cells	Rabbit model of defect at the fusion site of femur, vertebral cavity and posterolateral spine	[42]
OPF	BPNs	Controlled degradation rate, improving the spread, distribution, proliferation and differentiation of MC3T3 cells on hydrogels, and controling the cytotoxicity	–	[43]
OPF/ Collagen	BPNs	The appropriate 3D microenvironment for MSC cell culture, providing clues for osteogenic differentiation	–	[44]
OPF	BPQDs	The smallest BPQDs, promoting the spread, distribution, proliferation and differentiation of MC3T3 cells	–	[45]
WW/RSF	BPQDs packaged with PLGA	Strong mechanical properties, inhibiting osteoclast differentiation, and showing photothermal effects on spinal metastases	Femur defect in rat model and tumor-bearing nude mouse model	[46]
DNA and 3D-printed PCL	Vegf-engineered BPNs	Sustainable delivery of growth factors, promoting the growth of mature blood vessels, and inducing osteogenesis	Rat model of cranial defect of critical size	[47]
GelMA	BP@Mg	Biomimetic periosteal structures, significantly promoting angiogenesis by inducing endothelial cell migration, and upregulating the expression of neuro-associated proteins in neural stem cells (NSCs)	Rat model of skull defect	[48]
PVA and Chitosan	MgO blended BPNs	Excellent antibacterial effect, promoting the recruitment, osteogenic differentiation and biologic mineralization of MSCs	Rat model of skull defect	[49]
PLGA	BP-SrCl_2_	Excellent biodegradability, and photo-controlled Sr release	Rat model of femur defect	[50]

### Bone tissue engineering

In addition to the participation of osteoblasts and osteoclasts, bone mineralization induced by appropriate calcium and phosphorus metabolism is essential for bone remodeling. BP is highly homologous to the inorganic constituent of bone [50, 51]. The degradation product of BP is non-toxic phosphates. Phosphate is essential for bone regeneration [52] and may be utilized as a biomineralization inducer. Phosphate ions (PO_4_^3−^) serve as anionic ligands for positive calcium ions (Ca^2+^), which may stimulate the attraction, binding, and accumulation of free calcium ions in the physiological environment of bone tissue [53], resulting in the formation of calcium phosphate (CaP) nanoparticles. Calcium phosphate may facilitate the formation of hydroxyapatite (HAP), an essential structural component of bone and tooth enamel. BP regulates osteogenesis, osseointegration, and fracture repair due to its capacity to release phosphate ions and trap metal ions such as calcium [54].

Based on the above properties, Huang et al. [36] developed a BP nanosheets-based hydrogel platform by photo-crosslinking of gelatin methacrylamide, BPNs, and cationic arginine-based unsaturated poly (ester amide)s. Prior to ultraviolet (UV) irradiation, BP nanosheets (BPNs) were introduced to and mixed with the precursor solution. In addition to their gelling effect, cationic unsaturated arginine-based poly (ester amide) s [U-Arg-PEA] offered a positive charge to facilitate the hydrogel and BPNs’ strong electrostatic contact. Complementing one another, the surface charge and large surface area of BPNs fostered robust interactions with polymer chains and further reinforced the cross-linking network generated by UV irradiation. The addition of BPNs raised the hydrogel’s compressive modulus by three to four times, and it was biocompatible. hDPSCs cultured on the hydrogel surface were able to multiply and spread properly. This sustained supply of calcium-free phosphorus hydrogel approach was capable of releasing phosphate in response to light, accelerating mineralization in vitro, and promoting the osteogenic differentiation of hDPSCs through the BMP-RUNX2 pathway. In vivo studies from a model of a skull lesion in a rabbit shown that BPNs accelerated bone repair. Xu et al. [37] prepared scaffolds by loading BPNs and deferoxamine (BPN-DFO) into gelatin hydrogels using a similar approach. In ischemic tibial areas of SD (Sprague Dawley) rats with acute femoral artery blockage, the scaffolds displayed excellent swelling, degradation, and release rates, and significantly enhanced osteogenesis and blood vessel development.

In addition to BP/Gel-like nanocomposite hydrogels, Miao et al. [38] devoted particular emphasis to the exceptional photothermal performance of BPNs in comparison to GO and nanosilicate. The photothermal ablation of bacteria and tumor cells is enabled by the high photothermal conversion efficiency of BPNs [26, 55, 56]. The findings of their experiments indicated that BP/Gel nanocomposite hydrogels had excellent near-NIR photothermal property and the capacity to destroy cancer cells and germs. In vitro, the nanocomposite matrix may sustain cell proliferation and induce osteogenic differentiation of human mesenchymal stem cells (hMSCs) in the absence of bone inducible factor. In addition to the bactericidal and cancer cell-killing capabilities of BP, NIR irradiation enhanced the degradation of BP to PO _4_^3−^, increased the chemical activity, expedited the interaction between PO _4_^3−^ and Ca ^2+^, and promoted in situ biomeralization. In BP/agarose hydrogel [39], BP/chitosan/collagen hydrogel [40], and BP/PLGA hydrogel [41], it was shown that this property may be exploited to remotely regulate activation to accelerate biomineralization during bone tissue regeneration. This property may also be exploited for remote infrared controlled medication release. The mechanism of osteogenesis has also been examined further. BPNs may generate moderate photothermal effects induced by NIR light, and promote osteoblast recruitment via activating matrix metalloproteinase (MMP) and ERK-Wnt/β-catenin-RUNX_2_ axis mediated by heat shock proteins (HSPs) [57]. Some researchers in the field of bone tissue engineering have focused on the conductivity of BP [58]. Liu et al. [42] reported a new injectable carbon nanotube (CNT) and BP gel for tissue engineering with improved mechanical strength, conductivity, and continuous phosphate ion release. As a crosslinking matrix, a biodegradable oligomeric (poly (ethylene glycol) dimethyl fumarate) (OPF) polymer was used, and a crosslinked CNT-poly (ethylene glycol) -acrylate (CNTpega) was included to offer mechanical support and conductivity. However, CNT inclusion was primarily responsible for the hydrogel’s conductivity, and the influence of BPNs is minimal.

The appropriate concentration and size of BPNs in these novel nanocomposite hydrogel materials for bone regeneration need more investigation. In research, the addition of BPNs into a cross-linked OPF hydrogel improved the adhesion, distribution, proliferation, and differentiation of MC3T3-E1 cells. Optimal cell development was observed at BP doses up to 500 ppm [43]. In an additional research of 3D mesenchymal stem cell (MSC) preparations, Li et al. [44] examined the osteogenic response of MSC spheroids by a novel combination of collagen and BP. The stiff, wrinkly structure of BP offered mechanical signals for MSCs, which created a favorable microenvironment for osteogenic differentiation [59].

With 6 μg/mL collagen and/or BP concentration gradients (0 μg/mL, 4 μg/mL, 8 μg/mL，and 16 μg/mL), MSC spheres were effectively constructed. Runx2, osteopontin, and alkaline phosphatase were expressed at greater levels in the BP group with 4 μg/mL and 8 μg/mL concentrations than in the control group. Furthermore, the aforementioned findings showed that BP hybrid hydrogel might serve many functions in the cell treatment of bone regeneration, including precursor cell culture in vitro and carrier in vivo. In terms of BP size, the nanosheet form of BP is cytotoxic under certain circumstances [60], which restricts its biological applicability to some degree. The BP quantum dot is a novel kind of BP nanostructure that was initially created using the liquid exfoliation approach in 2015 and has a nanometer-scale dimension [61]. This ultra-small nanomaterial exhibited reduced cytotoxicity and more biocompatibility than BPNs [62]. Xu et al. [45] examined the osteogenic potential of BPNs of various sizes and quantum dots in BP/OPF hydrogels, where BP quantum dots was obtained by the combination of bath-sonication and probe-sonication. BPNs, particularly BP quantum dots, increased the behavior of MC3T3 cells on OPF hydrogels, including their spreading, distribution, proliferation, and differentiation. However, the intrinsic instability of BP quantum dots (particularly after being exposed to complicated blood circulation and physiological settings) is a primary barrier to clinical use. In order to solve this problem, Hu et al. [46] used the oil-in-water emulsion solvent evaporation method to encapsulated BPQDs into PLGA (poly (lactic-co-glycolic acid) to prepare BPQD/PLGA NS. Due of the hydrophobic structure’s special encapsulating effect, PLGA prevented the oxidation and degradation of BPQD by isolating internal BPQD, hence enhancing photothermal stability [63]. The combination of BPQD/PLGA NS and highly strong regenerated silk fibroin based delignified wood hydrogel increased the proliferation, migration, and osteogenic differentiation of BMSCs significantly. They were also surprisingly discovered that BPQD/PLGA NS inhibited osteoclast differentiation. Moreover, BPQD in hydrogels shown remarkable performance in vitro and in vivo for photothermal tumor ablation, giving evidence of potential therapeutic uses for both bone regeneration and bone metastasis ablation. These results give crucial references for the future selection of BP concentration and size in hydrogel scaffolds for bone tissue creation.

Due to their large specific surface area, BPNs are good drug carrier (drug loading efficiency up to 95%), and medicines are adsorbed on BPNs through non-chemical bonding (this property preserves drug activity and purity) [50]. Miao et al. [47] developed a dynamic DNA hydrogel modified with BPNs and VEGF to impart mechanical strength, where BPNs were modified by VEGF. Due of the non-covalent interaction between VEGF and BPNS, the integrated nanogel scaffold structure displayed a sustained VEGF release. In addition, BPNs were paired with a 3D-printed PCL scaffold to create a bioactive gel scaffold structure, in which BPNS attached to macromolecular DNA chains to impart function to the hydrogel and tighten the DNA cross-linking network. In vitro and in vivo, the whole gel scaffold system stimulated the growth of mature blood vessels and induced osteogenesis to encourage new bone formation. The capacity of BP to capture metal ions enabled it to transport helpful metal ions for bone rebuilding, such as magnesium.

Mg-doped biomaterials trigger bone regeneration by releasing Mg^2+^, therefore attracting MSCs and boosting osteogenic differentiation [64]. Recently, it has been reported that the modification of metal ion also considerably increased the stability of BP [65]. Xu et al. [48] developed a bilayer hydrogel that resembles periosteum to increase the efficacy of vascularized bone healing. The magnesium ion-modified BPNs (BP@Mg) was mixed into gelatin methacryloyl (GelMA) hydrogels to generate the top hydrogel as the periosteal repair layer, and the bottom hydrogel (GelMA-PEG/β-TCP) as the bone healing layer. The top hydrogel considerably increased angiogenesis by inducing endothelial cell migration and offerd many benefits for up-regulating the expression of neural-related proteins in neural stem cells (NSCs). This bilayer hydrogel approach opens the path for the development of biomaterials for neurovascular networks for bone regeneration. Magnesium may also be contained in hydrogels made by freezing and thawing processes by co-mixing with BPNs [49]. The results described above imply an optimistic future for BP-based nanocomposite hydrogels as therapeutic platforms for bone tissue engineering applications.

### Nerve tissue engineering

BP is mostly utilized for bone tissue engineering in the area of tissue engineering since its composition is close to the inorganic constituent of natural bone. However, investigations have shown that phosphate is also a crucial neurogenic differentiation signal promoter that may induce neurite outgrowth [66]. The availability of phosphorus ions, which are critical for regulating cell signaling, and the high conductivity of multiple electron pairs from phosphorus atoms in the BP fold layer may contribute to increased neuronal development [67, 68]. BP also has the potential to regulate the neural differentiation lineage of stem cells in stem cell therapies for neurological disorders. Strong electrical conductivity up to 300 S m^− 1^ [69] and good thermal conductivity may be exploited as a support to facilitate biological signal transduction, which may explain why BPNs are advantageous for promoting neural differentiation of stem cells. Xu et al. [70] have proven that the incorporation of polydopamine-modified BPNs into a hydrogel matrix significantly accelerated the differentiation of bone marrow-derived mesenchymal stem cells (BMSCs) into neurons in response to electrical stimulation. The rigidity of hydrogels and the conductivity of BP are regarded as optimal physical clues for regulating neuronal lineage control by MSCs. Modification or functionalization of BP is also a crucial element in stem cell differentiation regulation [71].

**Fig. 3:**
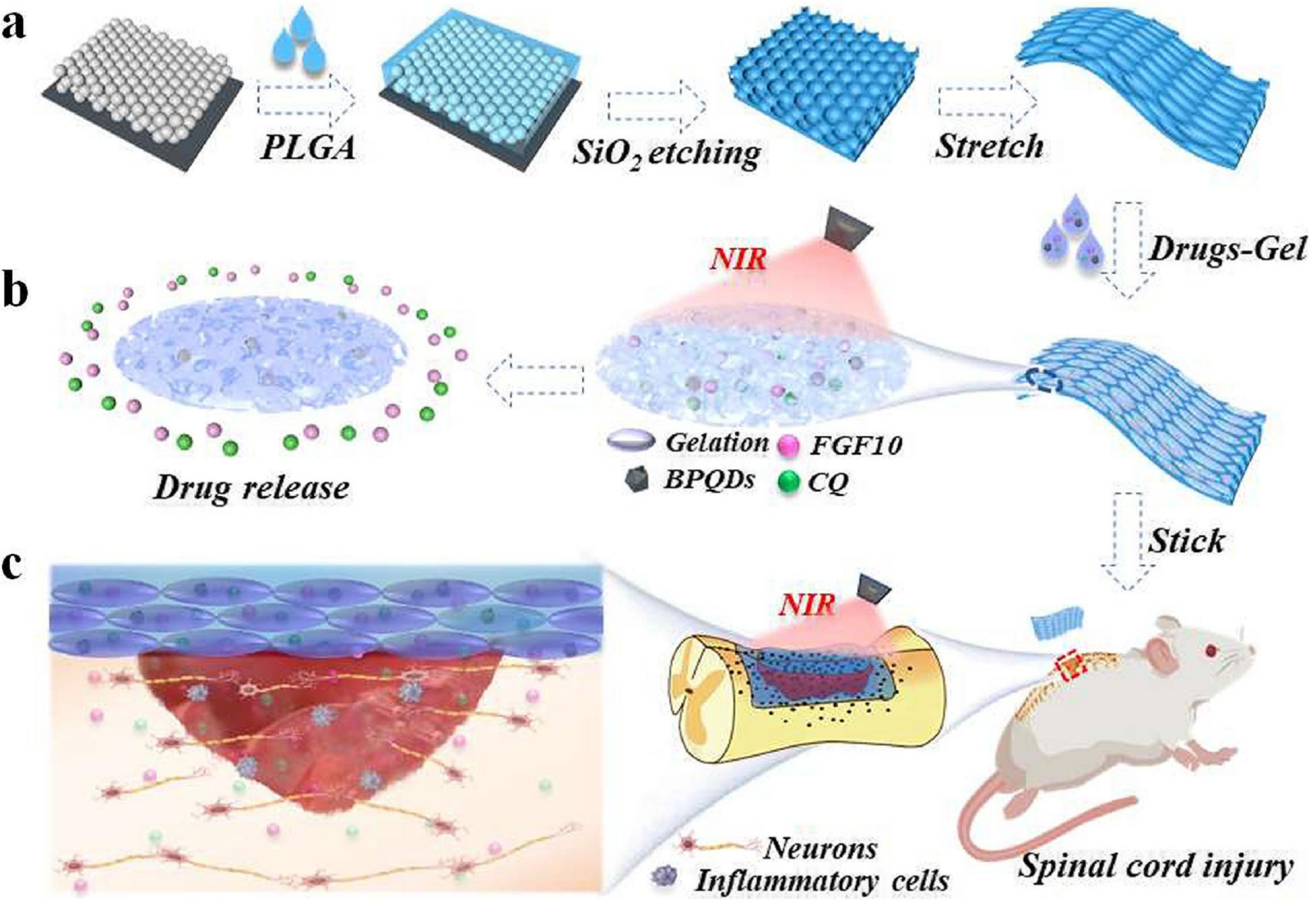
Schematic diagram of the fabrication of SIOF and its application in SCI repair. .Reproduced with permission from Ref [72]. Copyright 2019 Elsevier Ltd. All rights reserved

When designing engineered scaffolds for spinal cord injury healing, fundamental criteria such as structural stability, local microenvironment modeling, and cellular or biomolecular transmissibility should be taken into account. Under NIR irradiation, Wu et al. [72] employed BPQDs as a responsive drug release medium to produce hydrogels containing agarose, gelatin, and hyaluronic acid. Then, they permeated a temperature-sensitive hydrogel containing BPQD and drugs (fibroblast growth factor 10 (FGF10) and chloroquine phosphate (CQ)) into the pores of stretched inverse opal film (Drugs-Gel@SIOF), achieving Nir-controlled drug delivery and to a certain extent site-specific SCI repair in a rat model of SCI (see Fig. 3).

Recent research shown that BP promoted nerve regeneration by targeting copper ions, which are responsible for neuron degeneration in the blood-brain barrier (BBB). In addition to acting as nano-traps to control Cu^2+^ concentration, BPNs also lowered cellular reactive oxygen species (ROS) and protected cells from the toxicity associated with Cu^2+^ dysregulation [73]. Besides, the photothermal properties of BP confer exceptional permeability at the BBB, allowing the BP hybrid hydrogel with mechanical strength comparable to neural tissue to be utilized as a drug delivery and controlled release carrier in the central nervous system [73].

### Treatment of cancer

BP has a high absorption when exposed to visible light. The capacity of BPNs to generate ROS by absorbing light makes them good candidates for NIR-based photothermal therapy (PTT) and photodynamic therapy (PDT) for tumor. In addition to phototherapy, it has been shown that BP itself has an inherent chemotherapeutic effect [74] The photothermal antimicrobial capabilities and bacterial toxicity of BP are also advantageous in adjuvant antimicrobial therapy for the direct treatment of cancer-related infections [75, 76]. Besides, owing to their relatively wide surface area, BPNs have been used to load therapeutic agents in order to accomplish various therapeutic combinations, such as combined chemical/photothermal therapy, gene/photodynamic therapy, and others. Combined therapy for cancer has shown reduced adverse effects and increased therapeutic effectiveness [77]. The important property of bare BP is its propensity to oxidize (or deteriorate naturally) and precipitate in the tumor microenvironment, leading in a short treatment cycle and an uneven photothermal effect. Therefore, the development of anticancer nanocomposites based on BP remains difficult. Due to the benefits of minimally invasive, high local concentration, low systemic toxicity, local drug toxicity, and sustained release exclusively at the tumor site, appropriate injectable hydrogel carrier system forms have been the subject of intense study [78, 79]. Various smart hydrogel delivery methods, such as heat-sensitive, pH-sensitive, photosensitive, and dual-sensitive hydrogels [80], have been created in response to the various kinds and stages of cancer. This review discussed the use of BP composite hydrogels in the treatment of cancer (see Table 2).

**Table 2:** Application of black phosphorus composite hydrogel in cancer treatment

Hydrogel matrix	Type of tumor	Highlights	Application type	References
PLEL	HeLa tumor	High PTT efficacy and antimicrobial activity	PTT	[81]
Cellulose	Liver cancer. Melanoma	Excellent photothermal response, enhanced stability and good flexibility and biocompatibility	PTT	[82]
Agarose	Liver cancer	NIR controlled release of Emetine used to regulate SG formation in tumor tissues during PTT and improve tumor sensitivity to PTT	PTT/ Drug delivery	[83]
Polypeptide	Liver cancer	The temperature-sensitive system releases bufalin by light control, reducing its side effects	PTT/ Chemotherapy	[84]
OSA and AHA	Gastric caner	Photo-controlled release of paclitaxel effectively inhibits tumor cell proliferation	PTT/ Chemotherapy	[85]
Pluronic F127	Breast cancer	Photo-controlled release of gemcitabine with good photothermal efficiency and good biodegradability.	PTT/ Chemotherapy	[86]
PAHy	HeLa tumor	Excellent gel properties, pH sensitivity and NIR responsiveness.	PTT/ Chemotherapy	[87]
Pluronic F127	Breast cancer	DTX with particle size dominance is continuously released to generate ROS synergistic injury to tumors	PDT/ Chemotherapy	[88]
DNA	Breast cancer	Photo-controlled DOX release system with positive charge has high permeability, reduces drug resistance and improves survival	PTT/PDT/ Chemotherapy	[89]
Chitosan	Lung cancer, glioblastoma, liver cancer	Temperature-sensitive cavernous system, hemostasis, antimicrobial, high penetration of the hematologic tumor barrier, capturing CuNPs, utilizing CDT and PTT of CuNPs, synergistic with aPD-L1	PTT/CDT/IT	[90]
pNIPAM	Breast cancer, bladder cancer	Photo-controlled release of zoledronate as a scaffold for γδT cell proliferation and activation	IT/ Drug delivery	[91]
p (AAm-co-AAc)	Breast cancer, lung cancer	Pollen grains with their spike structure， high surface area, releasing cytokines and antibodies to proliferate and activate T cells	IT/ Drug delivery	[92]
Pluronic F-127	Lung cancer	Heat-sensitive, controlled release GM-CSF and LPS, personalized cancer vaccine, tumor antigen carrier, binding PD-1 antibody	IT/ Drug delivery	[93]

PTT is a novel kind of cancer treatment that destroys cancerous cells by the thermal action of exogenous light absorbers under NIR irradiation. Compared with other traditional treatment strategies, researchers have observed that PTT is easy, minimally invasive, has a low complication rate, and has a high spatial and temporal accuracy of near-infrared light, and diverse photothermal absorbers have been studied for PTT [94, 95]. Hydrogel platforms based on BP have also been employed for PTT. Shao et al. [81] exploited poly(d,l-lactide)-poly (ethyleneglycol)-poly(d,l-lactide)(PDLLA-PEG-PDLLA: PLEL) as heat-sensitive hydrogel matrix to develop a sprayable PTT system (BP@PLEL) by adding BPNs. BP@PLEL exhibited the potential to expedite the rapid transformation of sol-gel under NIR irradiation, obtain high PTT efficiency and antibacterial activity, which can be used to treat cancer postoperatively. Natural polymers may also be employed as host material for BP photothermal agent-based hydrogels. Xing et al. [82] developed cellulose and BPNs-based green injectable composite hydrogels (BPNSs). The green injectable composite hydrogel demonstrated an exceptional photothermal response, increased stability, and high flexibility on the basis of being fully non-toxic and biocompatible. In the PTT, a further rise in temperature causes in significant tissue damage. Inadequate heating of deep tumor tissue might result in tumor recurrence. Changing the sensitivity of tumor cells to PTT might potentially resolve this issue. Xie et al. [83] discovered that stress particles (SG) played a crucial role in the integration of internal and external stressors to control cell viability. SGs promoted and contributed in tumor resistance to PTT through a PTT-dependent mechanism reliant on eukaryotic initiation factor 2α. On the basis of this, they developed a hydrogel with BP as the photothermal agent for tumor-specific delivery and photo controlled release of NIR (SG inhibitor Emetine). Under near-infrared illumination, photothermal conversion of BPNs resulted in photothermal therapy (PTT) for tumor. Simultaneously, the photo-controlled release of Emetine in tumor tissues effectively blocked the synthesis of SG caused by PTT, rendering tumors susceptible to PTT, and so augmenting the tumor-inhibiting effect of PTT.

Due to their ability to absorb anticancer medications, BPNs can also be used to combine photothermal therapy with chemotherapy. Simultaneously, the intelligent co-delivery system of hydrogel can address the fundamental issues of drug toxicity, local sustained release, and photo-controlled release. Myocardial toxicity, for instance, restricts the therapeutic usage of Bufalin in cancer. In order to address this issue, He et al. [84] developed a BP hybrid polypeptide thermosensitive hydrogel (BP-bufalin@SH), which exhibited quick and significant temperature increase and released bufalin by photocontrol, therefore significantly minimizing the adverse effects of bufalin. Destruction of mitochondrial transmembrane potential may result in the irreversible apoptosis of cancer cells.

Sang et al. [85] prepared OSA/AHA/BP/PTX hydrogel by combining oxidized sodium alginate (OSA), amylated hyaluronic acid (AHA), black phosphorus (BP), and paclitaxel (PTX) under physiological conditions utilizing the formation of Scheff base bonds, and the results demonstrated excellent photothermal effect and sustained release ability of PTX. In brief, photothermal therapy combined with chemotherapy has shown more antitumor activity than chemotherapy alone [86, 87].

BPNs has been discovered to be an effective photosensitizer. Under NIR laser irradiation, BPNs generate a substantial quantity of ROS to destroy tumors, and have been employed in photodynamic therapy (PDT) [96–98]. PDT may also be used with chemotherapy, which has been extensively studied for its ability to boost anti-cancer effectiveness and inhibit tumor development. Li et al. [88] developed an injectable thermoreversible hydrogel (BPNs/ DTX-M-Hydrogel) employing F127 as the hydrogel matrix to encapsulate BPNs and docetaxel (DTX) micelles in order to promote the accumulation of medicines in tumor tissues and enhance anticancer activity. The combination of PTT with PDT is a potential strategy for enhancing treatment effectiveness. Zhou et al. [89] developed highly permeable, photothermal, injectable, and positively charged biodegradable nucleic acid hydrogel (DNA-gel) nanoparticles to deliver the anti-cancer medication azithromycin by combining cationic polymer PEI with negatively charged BPQD to generate PEI@BPQD. In mice with orthotopic breast cancers, DNA-gel treatment significantly decreased drug resistance and enhanced overall survival (83.3%, 78 days) compared to DOX chemotherapy alone. In addition, the beneficial photothermal effect of BP may increase the permeability of the blood tumor barrier (BTB) and facilitate therapy [99]. Utilizing this property, Wang et al. [90] developed a new therapeutic nanocomposite from chitosan (CS) hydrogel coupled with BPNs and copper nanoparticles produced in situ (CuNPs). The hydrogels (CS@BPNSs@CuNPs) were obtained with a temperature-sensitive spongy condition and a coagulation index of 24.98%. The released BPNs@CuNPs generated ROSto kill infected invasive bacteria (98.1%) and inhibit local residual tumor cell regeneration (11.3%), and demonstrated a 19.6% penetration rate across the BTB for the treatment of brain tumors. Combining the hydrogel platform with aPD-L1 immunotherapy (IT) produced a synergistic therapeutic effect for tumor prevention.

In addition to the aforementioned strategies, BP hybrid hydrogels may be used in cancer immunotherapy [100]. IT refers to processes that enhance the host immune system’s ability to react to tumors and create long-lasting antitumor responses [101]. Diverse immunotherapies for cancer, including immune checkpoint blockade (ICB) therapies, cytokine therapies, cancer vaccines, and adoptive T-cell therapies, have been developed and have shown promising clinical efficacy [102]. Emerging interest is particularly focused on manipulating nanocomposites as immune adjuvants to form unique drug delivery systems required for the combination of phototherapy and immunotherapy to eliminate primary and metastatic tumor cells by inducing dendritic cell (DC) maturation and cytotoxic T-lymphocyte infiltration [103]. For example, Shou et al. [91] employed the excellent biocompatibility of BPQDs-doped pNIPAM hydrogel particles to prepare scaffolds for the proliferation of γδT cells. In terms of γδT cell activation and growth, BPQDs-doped pNIPAM hydrogel particles loaded with zoledronate performed very well. Their team developed hydrogel-integrated natural pollen grains as artificial antigen presentation scaffolds for T cell activation and proliferation in vitro 2 years later. BP was in the scaffolds and enabled NIR to stimulate T cells by triggering the release of cytokines and antibodies. In addition, the pollen’s characteristic spike shape and large surface area led to the formation of T-cell clusters and increased their local proliferation. After in vitro growth, activated T cells exhibited significant anticancer activity [92]. Additionally, BP hybrid hydrogels might be utilized to deliver cancer vaccinations. Ye et al. [93] prepared BP quantum dot nanovesicles (BPQD-CCNVs) covered with surgically excised tumor cell membranes. Due of the presence of patient-specific tumor antigens in the surgically excised tumors, they were loaded onto heat-sensitive hydrogels containing GM-CSF and LPS. Sustained release of GM-CSF from subcutaneously injected Gel-BPQD-CCNVs effectively recruited dendritic cells to capture tumor antigens. LPS stimuli and NIR irradiation enhanced the growth and activation of DCs, which subsequently migrated to lymph nodes to deliver antigen to CD8+ T lymphocytes. In addition, the combination of PD-1 antibody significantly increased the eradication of surgical residual and metastatic lung tumors by tumor-specific CD8+ T cells. Therefore, it indicates that in cancer immunotherapy, in vitro cultivation of therapeutic T cells and personalization of cancer vaccines should give more attention to the BP composite hydrogel material.

In brief, BP composite hydrogels show significant promise as a novel way to enhance postoperative treatment (antibacterial property and tissue regeneration) and different therapeutic approaches for a wide range of malignancies (including but not limited to PTT, PDT, chemotherapy, drug delivery, immunotherapy, and vaccines).

### Wound healing

The treatment of skin incisions, particularly chronic complicated incisions such as diabetic infection incisions, remains a significant challenge in regenerative medicine [104]. Single dressings are utilized in the clinic. There is a shortage of systemic, multifunctional wound dressings with high absorbability, form customization, quick self-healing, directing tissue regeneration, and restoring physiological function. Hydrogels offer better flexibility, exceptional elasticity, outstanding biocompatibility, a high-water content, and physiological environment sensitivity compared to other materials. Therefore, researchers have paid particular attention to hydrogels [105, 106]. In the hydrogel repair scaffold for wound healing, the antioxidant capacity of BP itself and the antibacterial ability of photodynamic treatment, which generates a significant quantity of ROS under NIR, have also been investigated in more detail [107, 108].

**Fig. 4:**
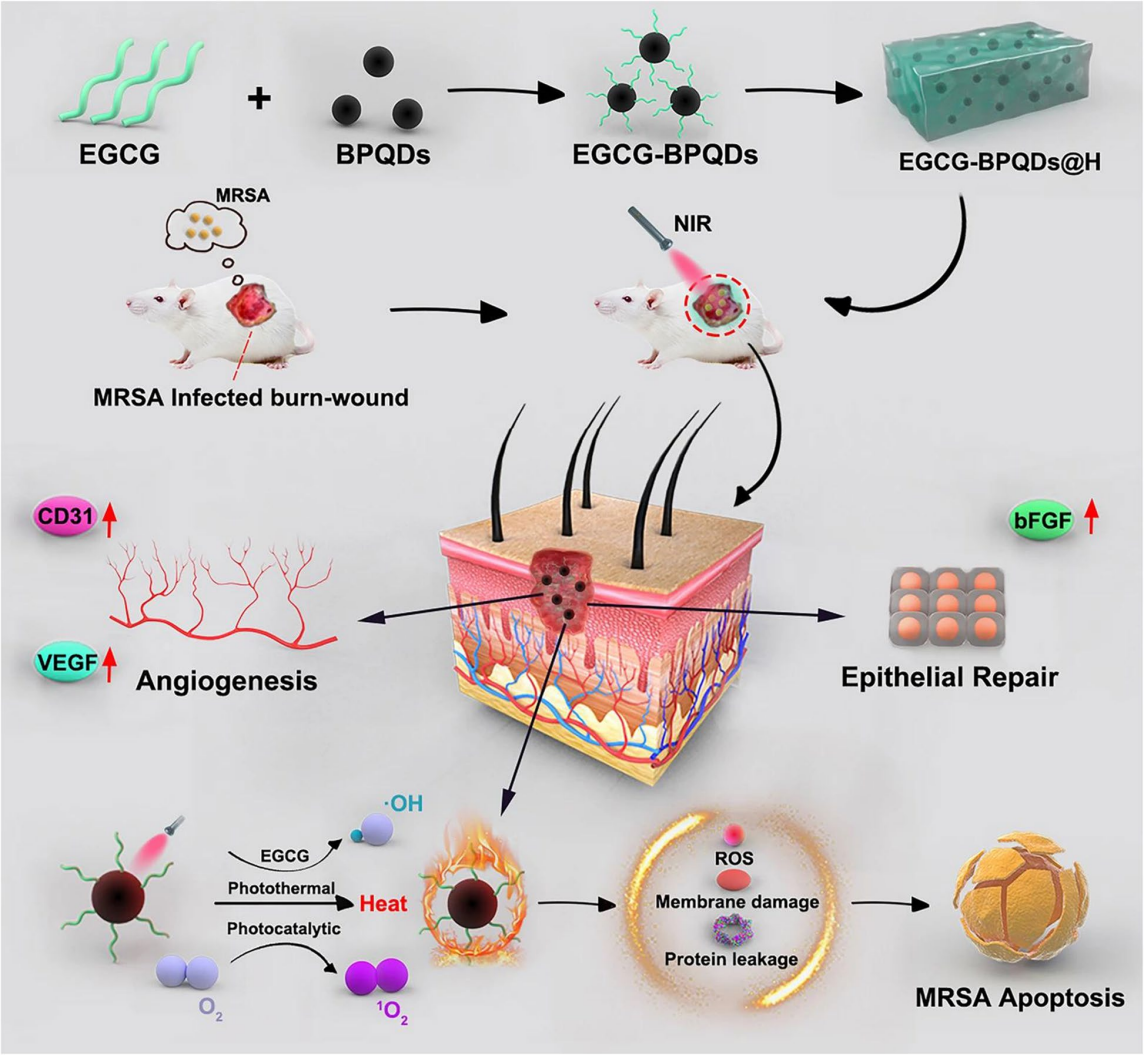
Schematic diagram of synthesis of EGCG-BPQDs@H nanocomposites and the process of bactericidal and stimulating cell behavior. Reproduced with permission from Ref [110] Copyright 2019 BMC Ltd. All rights reserved

Mao et al. [109] discovered that the hybrid hydrogel prepared based on the basic electrostatic interaction between BP and chitosan (CS) generated a substantial quantity of singlet oxygen (^1^O_2_) when exposed to NIR light, which killed 98.90% of escherichia coliand 99.51% pearl and 99.51% staphylococcus aureus within 10 minutes. In addition to enhancing the synthesis of early fibrinogen and accelerating the formation of scabs during tissue regeneration, the hybrid hydrogel was capable of repeatability. BPNSs progressively degraded to phosphate, activating the PI3K/Akt and ERK1/2 signaling pathways stimulating wound healing and bacterial infection, and enhancing cell proliferation and differentiation. Xu et al. [110] used EGCG (Epigallocatechin gallate) modified BP quantum dots to load onto berberine nanohydrogel (BNH) to form EGCG-BPQDs@H in the selection of healing platform for MRSA (methicillin-resistant *Staphylococcus aureus*) -infected deep burn wounds (MIDBW) in diabetic patients (see Fig. 4).EGCG-BPQDs@H was more essential than BPQDs@H for photocatalytic singlet oxygen generation, according to the findings of electron spin resonance. Inhibition data indicated that the GCG-BPQDs@H sterilizing rate against MRSA was 88.6%. According to molecular biological investigation, EGCG-BPQDs significantly raised CD31 nearly 4 times and basic fibroblast growth factor (bFGF) nearly 2 times, which was advantageous for promoting the proliferation of vascular endothelial cells and skin epidermal cells. Under NIR irradiation, the MIDBW area treated with GCG-BPQDs@Hwas promptly sterilized by heating to 55 °C. After 21 days of therapy, the MIBDW closure rate was 92.4%, which was significantly higher than the control group (61.1%). Huang et al. [111] attached BPQDs to PVA (polyvinyl alcohol) and Alg (sodium alginate) matrices to generate BPQDs@NH using the same MIDBW model. Under NIR irradiation, BPQDs@NH generatedROS, lipid peroxidation, glutathione, adenosine triphosphate buildup, and bacterial membrane breakdown, all of which killed MRSA in a synergistic manner. In addition, animal experiments showed that BPQDs@NH effectively closed 95% of MIDWs after 12 days by lowering the inflammatory response and modifying the production of vascular endothelial growth factor (VEGF) and basic fibroblast growth factor (bFGF). To improve the antibacterial and antioxidant properties of BP composite hydrogels, researchers have also explored synergistic effects with other substances with excellent antibacterial properties, such as antimicrobial peptides, metal ions (e.g., zinc ions and copper ions), anthocyanins with potent antioxidant properties, and 4-octyl itaconate (4OI), as well as with immunotherapy [112–115]. Trace zinc, for instance, inhibited the polarization of macrophages towards the M2 phenotype. A significant number of M2 macrophages release anti-inflammatory proteins and cytokines to decrease inflammation and promote neovascularization [114]. An additional instance is the production of dendrimer-modified X+ Y-type DNA joint structural hydrogel based on the base pairing principle, which supported the change of macrophages from the pro-inflammatory M1 to the repair M2 phenotype and maintained a stable wound remodeling state. Surprisingly, DNA-hydrogel dressings induced neurons to enter a repair state, hence speeding nerve regeneration and angiogenesis in the skin. In addition, it might recruit bone marrow cells to trigger adaptive immune responses and enhance DNA-hydrogel dressings’ potential to induce tissue regeneration, ultimately enhancing hair follicle and hair regeneration [115].

BP can also be loaded onto microneedle hydrogel tips for use in other wound healing attempts. The photothermal effect of BP is projected to dissociate oxygen from hemoglobin, therefore inducing local oxygen supply to increase skin cell proliferation and tissue remodeling [116]. It has also been shown that BP composite hydrogels generated in conjunction with gelatin or collagen matrix are a multifunctional and effective platform for the simultaneous treatment of melanoma and skin defect regeneration, which may cooperate with drug delivery and chemotherapy [117, 118].

### Other biological applications

As a new type of drug delivery carrier, 2D BP nanomaterial demonstrates a high drug loading capacity owing to its rippled crystallization and structural properties, and BPNs have the ability to generate ROS by absorbing light, implying a significant development potential in other biomedical fields. Pan et al. [119] developed a unique therapeutic platform for the treatment of rheumatoid arthritis by mixing BPNs with platelet-rich plasma (PRP) -chitosan thermos-responsive hydrogel. Under NIR irradiation, BPNs created local heat while providing ROS to the inflamed joint in order to eliminate hyperplastic synovial tissue. Moreover, PRP efficiently increased the adherence of MSCS to thermosensitive chitosan hydrogels. This heat-responsive hydrogel also preserved articular cartilage by minimizing tissue friction. Assays of methotrexate release and absorption revealed the drug’s delayed release qualities, and the whole system significantly decreased the degree of edema in mice with arthritic inflammation.

Intelligent drug delivery nano-systems react to tiny changes in the environment’s physical and/or chemical signals by significantly altering their physical and/or chemical properties and subsequently releasing medications suited to illness development at proper paces. Due to their unique properties in the use of intelligent drug delivery nano systems [120, 121], BP and other 2D materials have a major place in many scientific and technical domains. In a diabetic mouse model, Dong et al. [122] reported a novel real-time bidirectional blood glucose regulatory drug delivery system (BDRS) comprised of glucose-loaded pressure responsive nanovesicles (Glu@PRNV), insulin-loaded BNPs (Insulin@BPNs), hydrogels, and painless glucose monitoring patches. BDRS could monitor glucose levels in real time by observing color changes. Later, depending on the needs, BDRS could release glucose in response to external pressure or replenish insulin in response to NIR irradiation to precisely regulate blood glucose levels in diabetic patients within reasonable fluctuations, thereby reducing the likelihood of hyperglycemia or hypoglycemia. By incorporating BP hydrogel microspheres into microne (MN) arrays, Lu et al. [123] developed a novel approach to achieve multifunctional and controlled drug delivery. Solid MN arrays comprising BP and poly (N-isopropyl acrylamide) (pNIPAM) packed with porous ethoxylated trimethylol propane triacrylate (ETPTA) modulated blood glucose levels in streptozotocin (STZ) -induced diabetic mice through subcutaneously regulated insulin release. These findings suggest that with more study and in vivo testing in the future, the use of 2D stratified materials such as BP and its derivatives in intelligent drug delivery systems will be used in clinical practice.

## Concluding comments and future prospects

In comparison to other established nanomaterials based on hydrogels [124], the use of BP-based hydrogels remains in its infancy, and there are many research gaps. For instance, the study on the safety of 2D nanomaterials, particularly over the long term, is inadequate for biological uses. In addition, it remains difficult to develop easy, large-scale, cost-effective, and environmentally friendly production of BP composite hydrogel materials [125]. At present, bulk BP is produced mostly by converting white or red phosphorus under high temperature and pressure, with no water or oxygen required for the conversion. Consequently, the production of BP in bulk is restricted to the laboratory. In addition, the BP production is insufficient to fulfill the needs of future industrial applications. Therefore, there is an urgent need for a technique that can accurately manage BP characteristics, including size, number of layers, and surface modification, for the production of uniform BP. The particle size and its modification are crucial for controlling the biological behavior of BP [8]. The defining properties of bare BP is its vulnerability to oxidation (or natural degradation) and precipitation in the tumor microenvironment, which results in short-term therapy and uneven photothermal effects. Therefore, it is often required to develop a number of techniques to change and passivate the surface of BP in order to enhance its photothermal stability. During the antibacterial treatment of skin wounds, a substantial quantity of ROS generated by the photothermal effect of BP are toxic to the DNA of normal cells, leading to progressive oxidative damage and final cell death. Consequently, determining the appropriate dosage is essential. The behavior of BP in vivo and the method by which it interacts with diverse biomolecules also need further research. As regards toxicity, as research on BP is still in its infancy, theoretical and experimental studies on BP, such as the degradation process of BP in vivo, need to be enhanced. The key to the effective clinical use of nanomedicine is its in vivo safety. The primary component of BP material is phosphorus, which is an abundant element in the human body. Therefore, BP may be broken down into phosphoric acid in the human body and is non-toxic. In mice, the safety of BP was also shown. Long-term research is required to determine whether long-term usage may produce excessive poisoning of phosphate ions, such as loss of metal ions (such as calcium and magnesium) in the body. Modern biomedical technology, physicochemical technology, and precision manufacturing technology are required for inter-disciplinary research into the development of safe, dependable, effective, and low-toxic 2D materials with clinical applications.

Opportunities exist with challenges. Extensive experimental and theoretical research is currently ongoing to fully unveil the potentials of BP. Based on new technology, BP has been employed in many biomedical domains, however it has not been further investigated in combination with hydrogels. Examples include highly sensitive and specific medical sensors for diagnostic and prognostic monitoring, biological imaging based on the strong interaction between electromagnetic waves and BP crystal structures (photoacoustic imaging, thermal imaging, fluorescence imaging), drug delivery by multiple pathways, gene therapy, and others [126–130]. These directions may be used to hydrogels in the future. We believe that BP-based hydrogel scaffolds will become an important method for medical research and clinical treatment in the near future, bringing benefits to the diagnosis and treatment of patients with a variety of diseases, as a result of the in-depth study of their application in regenerative medicine and the resolution of in vivo stability and biosafety concerns.

## Data Availability

All data generated or analyzed during this study are included in this published article and its supplementary information files.
